# *Wolbachia*: a possible weapon for controlling dengue in Nepal

**DOI:** 10.1186/s41182-020-00237-4

**Published:** 2020-06-22

**Authors:** Sujan Khadka, Ram Proshad, Alina Thapa, Krishna Prasad Acharya, Tapos Kormoker

**Affiliations:** 1 Department of Microbiology, Birendra Multiple Campus, Tribhuvan University, Bharatpur, Chitwan 44200 Nepal; 2 State Key Laboratory of Environmental Aquatic Chemistry, Research Center for Eco-Environmental Sciences, Chinese Academy of Sciences, Beijing, 100085 China; 3 Key Laboratory of Mountain Surface Process and Ecological Regulation, Institute of Mountain Hazards and Environment, Chinese Academy of Sciences, Chengdu, 610041 China; 4 State Key Laboratory of Alpine Ecology and Biodiversity, Institute of Tibetan Plateau Research, Chinese Academy of Sciences, Beijing, 100101 China; 5 University of Chinese Academy of Sciences, Beijing, 100049 China; 6 Animal Quarantine Office (AQO), Budhanilkantha, Kathmandu, Nepal; 7 Department of Emergency Management, Patuakhali Science and Technology University, Patuakhali, Bangladesh

**Keywords:** Dengue, Dengue control, Nepal, Outbreak, *Wolbachia*

## Abstract

Dengue, a mosquito-borne viral infectious disease, causes a high morbidity and mortality in tropical and subtropical areas of the world. In Nepal, the first case of dengue was reported in 2004 followed by frequent outbreaks in subsequent years, with the largest being in 2019 taking the death toll of six. It is reported that the number of dengue fever cases are soaring in Nepal spreading from the plains to more hilly regions. This might have serious public health implications in the future when combined with other factors, such as: global warming, lack of early detection and treatment of dengue, lack of diagnostic facilities, poor healthcare systems and mosquito control strategies. Nepal, thus, needs a cost-effective mosquito control strategy for the prevention and control of dengue. The *Wolbachia*-mediated biological method of the dengue control strategy is novel, economic, and environment-friendly. It has been successfully trialed in several areas of dengue-prone countries of the world, including Australia, Malaysia, Vietnam etc. resulting in significant reductions in dengue incidence. Given the lack of effective vector control strategy and weak economic condition of the country along with the persistence of climate and environment conditions that favors the host (*Aedes* mosquito) for *Wolbachia*, this approach can be a promising option to control dengue in Nepal.

Dear Editor,

Dengue fever (DF) is the most common mosquito-borne viral infection caused by the dengue virus of Flaviviridae family and transmitted primarily by *Aedes aegypti* and *A. albopictus* [1]. There are four serotypes of dengue virus: dengue virus type 1 (DENV-1), dengue virus type 2 (DENV-2), dengue virus type 3 (DENV-3), and dengue virus type 4 (DENV-4) [1], and each serotype is associated with large-scale outbreaks causing serious public health emergencies [2]. The symptoms of the dengue range from non-specific viral syndrome (dengue fever) to fatal hemorrhagic disease (dengue hemorrhagic fever [DHF]) [2]. More than 3.9 billion people living in tropical and subtropical areas in over 128 countries are in risk of dengue [3], and each year around 100–400 millions of cases and several thousand deaths are experienced globally [1].

In Southern Asian countries over the past few decades, dengue virus has been reported with severe outbreaks as experienced in India [4], China [5], and other nations, showing that dengue is well established and is on the rise in neighboring countries. Nepal is no exception to this and has experienced several episodes of dengue outbreaks each year [6]. Due to the long porous border with India, there is always a high risk of the cross-country spread of dengue in Nepal [7] (Fig. 1). A study has reported that the nucleotide sequences of the Nepalese dengue strain are similar to those circulating in India suggesting that the dengue virus could have been introduced from India [8]. Nepal encountered the largest outbreak of dengue in 2019 which cost the lives of six people (Fig. 2) [9].

**Fig. 1:**
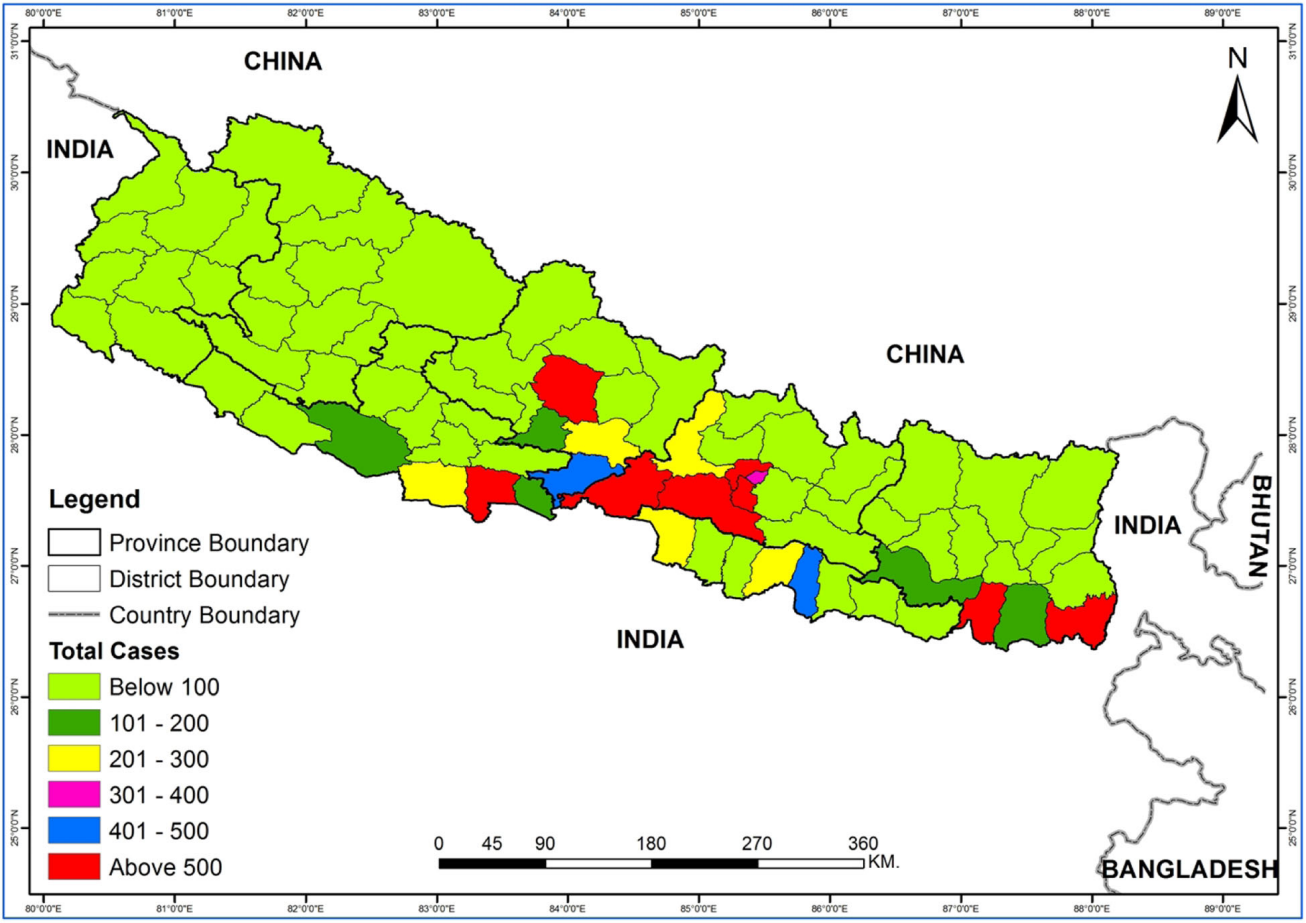
Map of the landlocked Nepal bordered by India and China and showing the total number of confirmed dengue cases reported from different districts of Nepal from fiscal year 2014/2015 until 2019/2020. Data were retrieved from the Epidemiology and Disease Control Division, Department of Health Services, Ministry of Health and Population, Government of Nepal

**Fig. 2:**
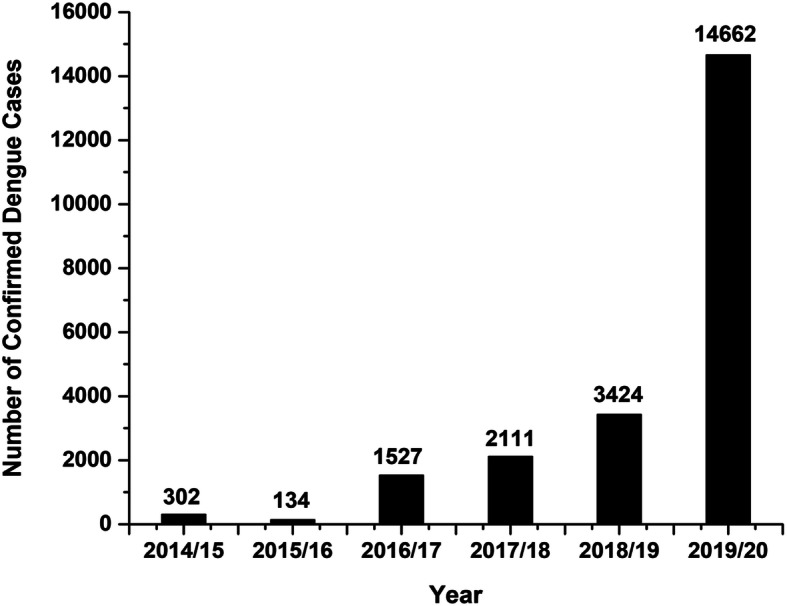
Confirmed dengue cases in Nepal recorded in each fiscal year starting from 2014/2015 until 2019/2020. Data were collected from Epidemiology and Disease Control Division, Department of Health Services, Ministry of Health and Population, Government of Nepal

The Ministry of Health and Population (MoHP) in Nepal has established an Early Warning and Reporting System (EWARS) to alert the healthcare systems on potential disease outbreaks [10]. However, EWARS failed to alert the healthcare systems concerning potential dengue fever outbreaks [6], and MoHP’s counterstrategies to cope with dengue incidence were insufficient. Lack of early detection and treatment of dengue cases, limited diagnostic tools, and poor healthcare systems are the major challenges to the fight against dengue in Nepal [11]. From the viewpoint of climate change, Nepal is one of the most vulnerable countries which is warming at a higher rate than the global average (1.5 °C in the last 25 years), and further, it is anticipated to be warmer and rainier in the future [12]. As a result, dengue is more likely to spread to higher elevations and outbreaks might escalate in the future [13–15]. Actually, such situation has already been evinced in the hilly and mountainous regions of the country (Fig. 1). This warrants for the need of developing an effective dengue control strategy taking into account the effect of climate change in the incidence of dengue in Nepal.

Until now, the main strategy employed for controlling dengue in Nepal has been reducing the abundance of the primary vector mosquito *Aedes* sp. using insecticides and clearing their breeding grounds [16]. However, lack of full effectiveness of the aforementioned methods, the emergence of insecticide resistance in vectors, and environmental pollution issues have motivated the search for newer approaches [17]. Whereas biological control strategies (such as use of fish, copepods, spiders and geckos) have some promising results in the control of mosquito-borne diseases including dengue in Vietnam [18, 19], these methods are associated with a high cost, requiring constant intercessions, and are unlikely to be introduced in peri-urban and urban communities [18, 20]. Some microbial bio-insecticides such as *Bacillus thuringiensis* subsp. *israelensis* (BTi), an entomo-pathogenic bacterium, have been proven successful to eliminate mosquitoes with effective larvicidal activities [21]; however, a short residual activity has reduced its efficacy in controlling mosquito-borne diseases [22]. Considering the circumstances, there is an urgent need of novel, cost-effective, and eco-friendly strategies to control dengue, which could be possible by using *Wolbachia*, an intracellular Gram-negative bacterium.

After the first introduction of *Wolbachia* in 1923, this perceived as being successful in controlling the population of harmful arthropods due to its effective vertical transmission and ability of replication inside the arthropods (Fig. 3) [25, 26]. The secrets of *Wolbachia* success can be seen by its ability to infect both male and female mosquitoes. Infected female mosquitoes lay eggs that harbor *Wolbachia*, and when *Wolbachia*-free females mate with *Wolbachia*-infected males, the eggs do not hatch, thereby limiting the vector proliferation (which is crucial for controlling dengue) (Fig. 3) [23]. Moreover, once introduced into mosquitoes, *Wolbachia* interferes with pathogen transmission and shortens the core lifespan of the host [24, 27]. Mosquito is crucial in the transmission of dengue virus, as after the entry in the mosquito’s body through an infected blood meal, dengue virus needs 8–12 days to spread on the mosquito’s body before migrating to the host’s salivary glands so it can be transmitted to the healthy humans [28]. An infected mosquito can transmit dengue virus for its entire lifespans lasting for 3–4 weeks in general [28]*.* Furthermore, when a *Wolbachia*-infected male mates with a female that is uninfected or harboring a different *Wolbachia* type, it induces cytoplasmic incompatibility (CI), also known as reproductive abnormality, resulting in early embryo death of the host [29]. *Wolbachia-*transfected mosquitoes experience upregulation of aae-miR-2940 in their cell lines, targeting metalloprotease genes and eases colonization of bacterium inside the host [30]. Several consequences are observed after the establishment of *Wolbachia* inside the mosquito host which result in reproductive abnormalities such as modifications within male sperm, feminization, and developmental disorders by altering the nutritional uptake in the mosquito (Fig. 3) [24]. *Wolbachia* indirectly nullifies the dengue virus living inside the host by ROS Toll pathway [24, 31] and exploits the miRNA of the host by altering the expression of methyltransferase gene DnmmtA2, thereby inhibiting the viral replication and enhancing its establishment within the cells of vector mosquito [24]. This inhibition of replication of dengue virus along with the propagation within the *A. aegypti* and *A. albopictus* populations is effective in either partially or completely blocking virus transfer to humans, which has, thus, a vital role to halt the transmission of dengue virus.

**Fig. 3:**
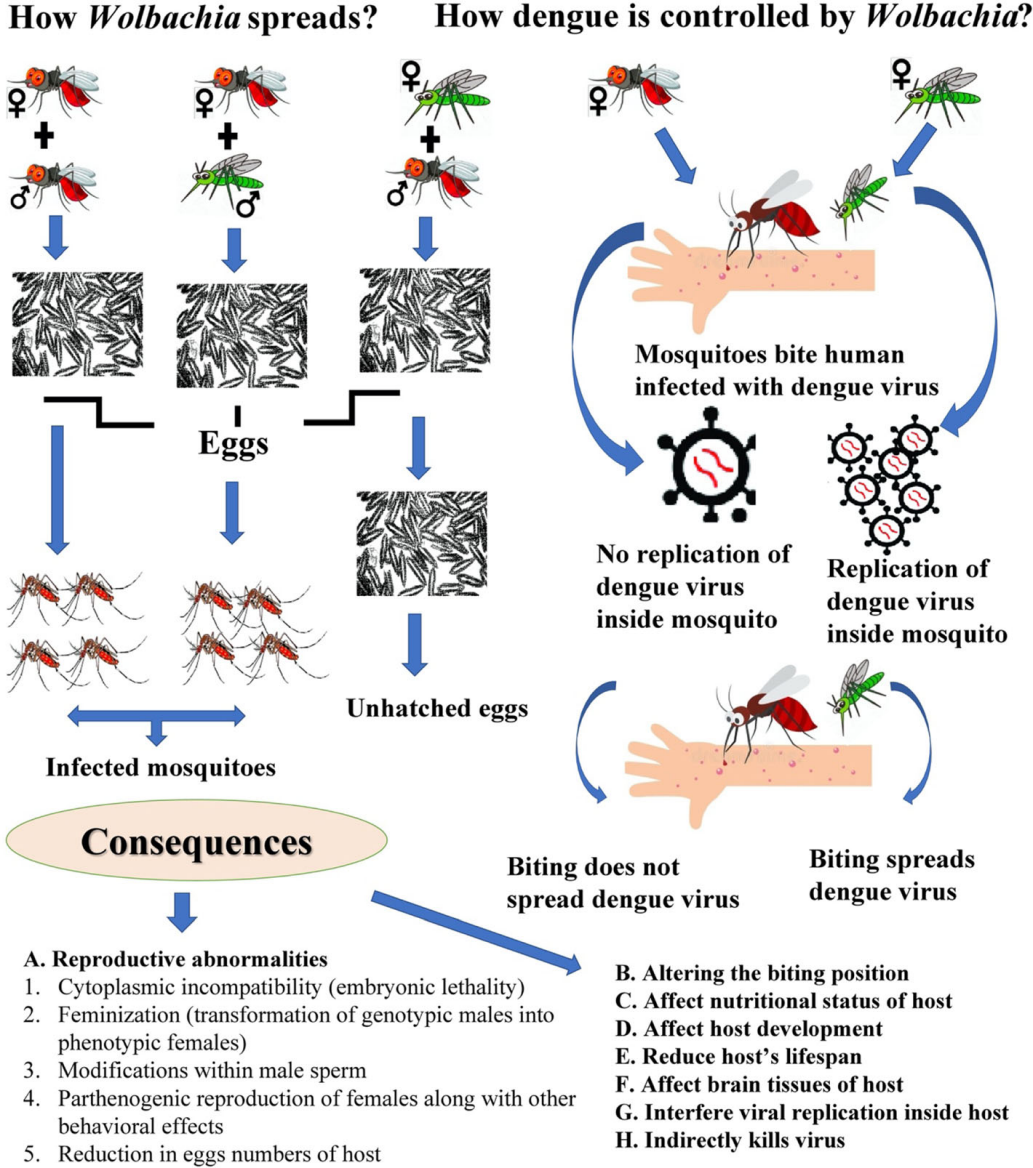
Vertical transmission of *Wolbachia*, its role in controlling dengue [23], and consequences within the infected host [24]. Note: red colored mosquitoes are infected with *Wolbachia* while green colored mosquitoes are uninfected. ♀ indicates female mosquito and ♂ symbolizes male mosquito

Different strains of *Wolbachia* have already been trialed in many dengue-affected areas of other countries (such as Australia [32], Malaysia [33], and Vietnam [34]) where the results were primising. For example, in a study at Belgian Gardens—an inner coastal suburb in the City of Townsville, Queensland, Australia, after the release of four million *Wolbachia*-infected mosquitoes in 2014, nearly 100% mosquitoes were infected with *Wolbachia* after 1 year and the cases of dengue plummeted drastically [32]. Likewise, in another trial conducted by releasing approximately half-million of *Wolbachia*-infected mosquitoes in Vinh Luong (a crowded dengue-prone district in southern Vietnam), cases of dengue were reduced by 86% since the trial [34].

The climate in Nepal varies with its topography and altitude with monsoon season starting from June and lasting until August. During this season, rainfall deposits the water in household containers, ditches, old automobile tires, buckets, blocked gutters, trash, buildings under construction, etc., creating a favorable environment for the multiplication and proliferation of *Aedes* mosquito [16]. Since the stable population of the host (*Aedes* sp.) (needed for proper multiplication of *Wolbachia*) are abundantly distributed in most of the areas in Nepal [35], several strains of *Wolbachia* can be effectively used to infect mosquito population with minimal cost. Apart from its cost-effectiveness, *Wolbachia*-mediated control method is eco-friendly [24]; therefore, it is one of the best possible options for low-income and resource-poor countries like Nepal. Although the initiation of this *Wolbachia*-mediated control method in Nepal might decrease its reliance on currently employed dengue control methods such as clearing the mosquito breeding sites, using anti-mosquito strategies (using mosquito nets and repellents, applying screens on windows and doors to stop mosquitoes from entering the house, wearing long-sleeved and light-colored clothes, and using household insecticide aerosol, mosquito coils, and vaporizers), and public awareness program in control of dengue, these techniques should not be discouraged in the initial stage of this new technique, which might jointly help to tackle the problem of dengue in Nepal. However, to implement this strategy, there is the need for the Government of Nepal to develop and prioritize *Wolbachia-*mediated dengue control including the (1) development and establishment of effective laboratory networks to early diagnose, detect, quickly respond, and report dengue; (2) development of qualified manpower to perform surveillance, track, check, and carry out data analysis to determine dengue-affected areas; (3) establishment of laboratories to grow *Wolbachia*-infected mosquitoes; and (4) inculcating sanitary habits and maintaining a clean environment.

Controlling dengue in Nepal is challenging yet possible. It requires a high degree of scientific, political, social and economic commitment. *Wolbachia* could be the best possible eco-friendly weapon for controlling dengue in Nepal posing no direct risk to human and environmental health.

## Data Availability

Data included in Figs. 1 and 2 are retrieved from the Epidemiology and Disease Control Division, Department of Health Services, Ministry of Health and Population, Government of Nepal. Dengue Control Program [Internet] [cited April 2, 2020]. Available from http://www.edcd.gov.np/section/dengue-control-program.

## Abbreviations

BTi: *Bacillus thuringiensis* subsp*. israelensis*
CI: Cytoplasmic incompatibility
DF: Dengue fever
DHF: Dengue hemorrhagic fever
DENV: Dengue virus
MoHP: Ministry of Health and Population
EWARS: Early Warning and Reporting System
